# Coordinated analysis of exon and intron data reveals novel differential gene expression changes

**DOI:** 10.1038/s41598-020-72482-w

**Published:** 2020-09-24

**Authors:** Hamid R. Eghbalnia, William W. Wilfinger, Karol Mackey, Piotr Chomczynski

**Affiliations:** 1 University of Wisconsin-Madison, Madison, USA; 2 University of Cincinnati, Cincinnati, USA; 3 Molecular Research Center, Inc., Cincinnati, USA

**Keywords:** Computational biology and bioinformatics, RNA sequencing

## Abstract

RNA-Seq expression analysis currently relies primarily upon exon expression data. The recognized role of introns during translation, and the presence of substantial RNA-Seq counts attributable to introns, provide the rationale for the simultaneous consideration of both exon and intron data. We describe here a method for the coordinated analysis of exon and intron data by investigating their relationship within individual genes and across samples, while taking into account changes in both variability and expression level. This coordinated analysis of exon and intron data offers strong evidence for significant differences that distinguish the profiles of the exon-only expression data from the combined exon and intron data. One advantage of our proposed method, called matched change characterization for exons and introns (MEI), is its straightforward applicability to existing archived data using small modifications to standard RNA-Seq pipelines. Using MEI, we demonstrate that when data are examined for changes in variability across control and case conditions, novel differential changes can be detected. Notably, when MEI criteria were employed in the analysis of an archived data set involving polyarthritic subjects, the number of differentially expressed genes was expanded by sevenfold. More importantly, the observed changes in exon and intron variability with statistically significant false discovery rates could be traced to specific immune pathway gene networks. The application of MEI analysis provides a strategy for incorporating the significance of exon and intron variability and further developing the role of using both exons and intron sequencing counts in studies of gene regulatory processes.

## Introduction

High-throughput RNA sequencing (RNA-Seq) has rapidly become a default standard for profiling the composition of the transcriptome. The starting material for RNA-seq is usually total RNA consisting of a heterogeneous pool of RNA molecules at different levels of gene transcript processing^1^. Profiling and subsequent differential analysis is routinely performed by measuring exon expression levels for the coding sections of an RNA transcript. A range of statistical and computational approaches have been developed to perform differential analysis^2–11^. However, RNA-Seq data also contains information about the intronic part of a protein-coding gene transcript^12^. While cells are known to contain substantial amounts of intronic RNA originating from unprocessed (primary) gene transcripts^13^, routine expression analysis does not study this data. As a result, an estimated 20–40% of sequenced RNA that is mapped to functionally relevant introns remains relatively unexplored^14–19^. Several approaches for identifying and profiling introns have been developed, but the topic of how to use the information remains the focus of intense research^20–25^. Characterization of intronic read data appears to be central to further progress^26–28^ (see also the guidelines of the Roadmap at https://www.roadmapepigenomics.org/).

The proportion of exons to introns in an unprocessed transcript is approximately 1–1 because both exons and introns are transcribed at approximately 1–1 ratio. Fundamental degradation kinetics suggests that free introns degrade according to the exponential decay law. Thus, the exon to intron relationship for each gene within and across samples might indicate comparative degradation rates across the genome. More broadly, evaluating the data regarding a gene’s joint exon–intron relationship may reveal how degradation rates in some genes are relevant to regulation. Related ideas have motivated the recent work on exon–intron relationship in RNA-Seq. Along these lines, investigators have estimated the change in mRNA half-life by using the difference of the logarithm of fold-change of exonic and intronic reads (ΔLog(exon)–ΔLog(intron)), and have suggested corrections for cases where the rate of transcription is too fast or slow^20,29,30^. Incorporation of intronic read data has also been used to determine RNA Velocity—the time derivative of gene expression^31^. These recent studies, performed in a variety of organisms, have delineated a new model in which for a subset of genes the most important regulatory sequences are located within introns.

When read counts are lower, as is the case for most introns, variability is a bigger barrier to attaining statistical significance. In addition to physiological variability, RNA purification, library amplification and the processing of RNA-Seq samples are among several factors that can introduce technical variability^32–34^. Other factors that introduce unwanted variability should also be mitigated to the extent possible. For example, blood-derived RNA is frequently used in studies of gene expression in humans, but in healthy individuals RNA content varies over a three to fourfold range^35^, and there is broad heterogeneity between individuals in the number and composition of blood cells which further contributes to biological variability^36–38^. When studying exon–intron relationships, each distinct experimental protocol carries with it a different set of trade-offs and variability profiles. For example, using poly-adenylated RNA for analysis can improve signals from mature RNA, but this may come at the expense of missing functionally important non-coding RNA fragments present in the nucleus and cytoplasm.

Computational tools play a key role in the analysis of RNA sequencing data, and standardization of computational workflows can mitigate the amplification of experimental variability. Currently, a variety of software platforms and workflow protocols are available for quantifying expression levels^2,4,5,8,10,11,39–45^. Quantification can be achieved by counting the number of RNA fragments 50–100 nucleotides in length (reads) that overlap with a sequence in the reference genome. Quantitation by counting the number of reads (Read number) provides an estimate of expression for complete mRNAs, individual exons and introns, and intervening lncRNA junctions. A significant number of these reads (> 20%) include counts of non-uniquely mapped reads that are multi-mapped to more than one sequence. Computational methods have been devised to distribute the multi-mapped reads between the various assignment locations in accordance with various statistical models. Assignment of multi-mapped reads, and general software assumptions can introduce consequential bias in both exon and intron reads^44–48^. Therefore, minimizing assumptions and simplifying computation is expected to mitigate the potential for bias in results.

In some studies, quantifiable changes in variability attributable to physiological conditions in control vs. disease have been identified as important predictors^49,50^. For example, differences in exonic expression entropy (after correction for technical variability) between control and case conditions, has been used as a differential measure for detecting change^51^. However, the broader use of measures that capture physiological variability of expression as a differential between disease and control has been otherwise rare and limited to exonic expressions. The recently established role of introns in regulation supports a more comprehensive approach towards investigating physiological changes by: (a) considering the joint characterization of exon and intron data in expression analysis, and (b) including changes in both expression level and expression variability. Genes with altered exon or intron expression, or altered exon or intron variability, can then be used to identify significantly altered gene expression pathways.

To this end, we introduce the Matched change characterization for Exons and Introns (MEI) methodology for examining simultaneous changes in the exon and intron counts, and their variabilities for all genes in one individual as well as one gene across individuals. Genes selected through MEI analysis can be used to identify significantly altered gene circuitry, which can in turn guide the development of new differential tests. The MEI methodology can reveal unique information not previously identified by current methods, and is applicable to a wealth of existing data in databases with relatively small computational overhead. To further improve the robustness and reproducibility of MEI, we employ a strict counting technique that is applicable to other RNA-Seq studies.

Our study uses genes with high-quality mapped reads exclusively—reads with high MAPQ values^40^—which makes the counts obtained for both introns and exons effectively “unique” counts. We refer to these counts as “singular counts” because they are effectively singularly aligned to only one gene. To improve the robustness of expression data, our in-house study employed ERCC spike-in controls for read calibration by implementing a proportional adjustment method that compensated for amplification and pipetting errors^52^. In addition, we introduce the rescaled exon to intron reads ratio for visualizing the differences between exons-only and exon/intron ratio data. The analytical MEI approach provides persuasive experimental evidence for the involvement of intra-gene intronic regions in the regulation of differential gene expression, and it also uncovered additional genes reaching statistical significance in the differential analysis of the two groups considered.

## Materials and methods

### RNA samples

This study was approved by the Chesapeake Institutional Review Board (CIRBI Protocol Pro00009509). All methods were performed according to applicable regulations and guidelines approved by the Chesapeake IRB committee. A total of 24 women ranging in age from 50–82 years of age (mean ± SD: 62.71 ± 9.37 years) and 11 men of age 52–89 years (64.27 ± 10.29 years) provided informed consent prior to participating in the study. Participants were interviewed by a medical professional and eligibility was based on a questionnaire containing 53 health-related questions approved by Chesapeake IRB. All participants were in good health at the time of blood collection and after an overnight fast, 8–10 ml blood samples were collected into two 10 ml BD Vacutainers (EDTA) by New Horizons Clinical Research, Cincinnati, OH. The blood from one Vacutainer was transferred into a pre-weighed bottle containing 16 ml of RNAzol-BD and the blood was thoroughly mixed with the reagent prior to storage at – 20 C. The second tube of blood was processed for Complete Blood Cell (CBC) analysis by LabCorp, Dublin, OH. A complete summary of the CBC data is available in a previous report^35^.

RNA was processed according to the manufacturer’s directions (RB-192; Molecular Research Center, Inc., Cincinnati, OH) and details were previously reported^35,53^. The solubilized RNA was analyzed with a Bioanalyzer (RIN = 7.43 ± 0.31, n = 35), and stored at – 80 C until the samples were thawed for library preparation. Prior to RNA-Seq analysis, samples were DNase-treated. Absence of gDNA contamination was verified through negative control PCR experiments. Additional details are provided in the supplementary material (Supplement S1). The DNase-treated samples were submitted to the University of Cincinnati Genomics, Epigenomics and Sequencing Core Facility for RNA sequencing. After the samples passed quality control analysis, an aliquot of ERCC ExFold RNA spike-in mix (Ambion, 4456739; Foster City, CA) was added to 1 µg of large RNA prior to rRNA and globin mRNA depletion with the Globin-Zero Gold Kit (Illumina GZG1206; San Diego, CA). The cDNA libraries were processed according to standardized Illumina protocols prior to sequencing on the Illumina HiSeq 2000 platform (see Supplement S8).

Additional RNA-Seq data from peripheral blood were obtained from the Sequence Read Archives (SRA) public repository (https://www.ncbi.nlm.nih.gov/sra). The data were part of the study by Mo et al.^54^ (https://www.ncbi.nlm.nih.gov/geo/query/acc.cgi?acc=GSE112057). We used the data for the 12 control and 46 polyarthritic samples provided in the case study to illustrate the use of MEI analysis.

### Workflow

Our workflow for RNA-Seq quantification (Supplement Fig. 1s) followed standard procedures^39,40^. As a first step, we evaluated the quality of raw data for adapter contamination, average base quality score per read, GC content distribution, and other relevant parameters. All samples were evaluated using FastQC^55^ software. Fastq files contained 53.9 to 77.1 million reads, and the initial QC result for the 35 samples was a 97.6% at Q score ≥ Q30 (probability of correct base assignment 999/1,000 times). The files were trimmed and processed in preparation for subsequent data analysis. Reads were aligned to the reference genome by using the BowTie2 aligner, which supports gapped alignments in the latest release^56^. FastQC software was also used to verify the quality of aligned data in files generated by Bowtie2. All of the sequencing data in this report were processed as single-end read counts. For the 35 control samples, ERCC spike-in counts were identified in the alignment step by augmenting the reference genome GRCh37.p13 [hg19] with ERCC spike-in^57^ sequencing data (provided by the manufacturer).

**Figure 1:**
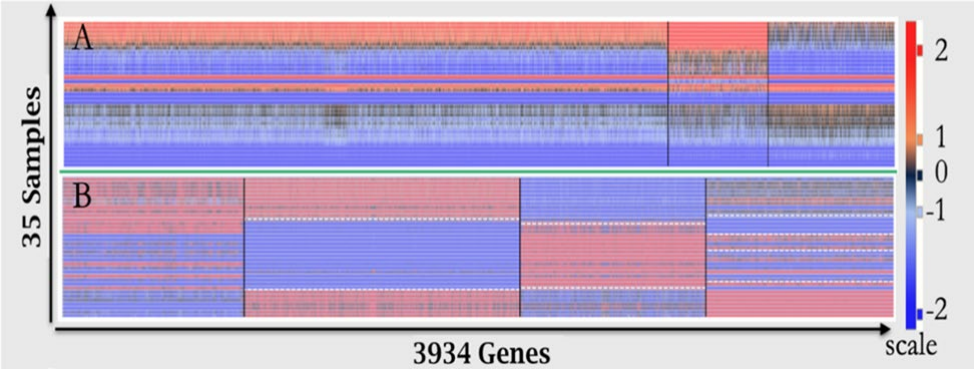
Clustering profiles of exon counts and exon/intron ratio data. The profile of each individual in the control group (n = 35) is depicted as horizontal slice (rows) of the heatmap. All values were transformed using log_2_ and range-normalized by using the z-score function (mean = 0). The normalized values for **A** and **B** are represented by the scale color bar on the right side of the heat map, with warmer colors (red or r) indicating a positive z-score and cooler colors (blue of b) indicating a negative z-score; z-score values near 0 are highlighted with black. Identical color scale is used for both heatmaps. In **A** and **B**, each vertical slice (column) represents the z-score profile of one gene across the 35 samples. Samples (horizontal rows) are organized according to correlation with highest correlated rows closer to each other. In **A**, three distinct clusters were formed for the exon-only data and more than 70% of genes were observed in the largest cluster. In **B**, the transformation and normalization of values was performed on exon/intron ratios. Four distinct clusters emerged in **B** (separated by black lines in the heat map). For example, the profile (row) of an individual near the bottom of **B** can be summarized as brbr (left to right dominant colors). The blue color scale in the last 7 rows of **A** (bbb) indicate below zero mean z-score values for all genes in these 7 individuals. No rows in **B** have a bbbb (or rrrr) pattern. Similar observations can be made for z-score values above zero in rows 20 and 22 of **A**. The horizontal dotted white lines in **B** are used as a visual aid to indicate the transition boundary. Note that the width of each cluster in **B** is adjusted for best presentation and also for comparison to the NCBI control data in the supplementary data (Supplement S4) and it is not strictly proportional to the number of genes in the cluster.

The aligned data files were used to obtain high-quality count data which represented singular gene counts (see Supplement S2). Bowtie2 quality flags were used to establish a threshold for singular counts. Genes qualified for singular counts satisfied two conditions: (a) we required a MAPQ ≥ 40, which meant that sequences were mapped with high probability to one location in the genome, and (b) if lower quality counts for the same gene or sequence were reported, we required the alternative counts not to exceed 1% of the total gene counts. The procedure detailing this selection criteria and its impact is provided in the supplementary material. Counts provided by two common software tools (Cufflinks and HTSeq^58^) were used as a quality check for the singular counts obtained with our in-house scripts. The validity of intron reads was further confirmed with quantitative PCR for a selected set of transcripts displaying different levels of gene expression (Supplement S1). PCR results confirmed the presence of intron sequences as measured by RNA-Seq results. For the set of 35 healthy subjects, we obtained singular counts for 4,865 genes. The NCBI data set yielded singular counts for 4,292 genes. We note that the singular count values do not directly address duplicated genes (genes that map to more than one location); of which there were 17 in our data set. We used counts for duplicated genes only if they had singular counts. Additional information is outlined in the supplementary material (Supplement S2).

The data for the 35 controls prepared for this study utilized external spike-in controls. A library of 96 external RNA spike-in controls, developed by the External RNA Controls Consortium (ERCC), was designed to act as a technology-independent control for differential expression experiments^52,57^. In order to evaluate and compensate for the effect of technical errors on the sequencing results, our computation incorporated ERCC spike-ins to build an iterative calibration model using the known ratios among ERCC concentrations in the original commercial mix (see Supplement S3 for additional discussion). The calibration step provided size factor correction, which was omitted in favor of correction to median^62^ when ERCC data was not available (see Supplement S1, Fig. 1s and discussion). The above protocols were chosen to minimize sequencing and mapping bias. The public data set did not yield any data for external spike-in controls.

### Computational analysis

We used GINI coefficients for evaluating variability^59^. The GINI coefficient, sometimes referred to as GINI, is a number between 0 and 1 with lower values indicating less variability. More accurately, GINI is a measure of heterogeneity or dispersion of data, and is related to entropy and higher statistical moments of data. GINI is obtained by normalizing the absolute value of the sum of differences between all expression values for any given gene sample. GINI is not impacted by the upper and lower range of data, it is not sensitive to data heteroscedasticity (overdispersion problem) or normalization effects, it works well even when the data is not normally distributed, and it is robust to outliers. For example, GINI for a set of gene-length-normalized counts is the same as the raw expression counts (not length-normalized). We use the unbiased formulation of GINI and note that differences in the number of samples, or read depth have minimal impact on GINI. GINI and Coefficient of variation (CV) are follow the same trends for normally distributed data without outliers. Since RNA-Seq expression data is not normally distributed and can contain outliers, GINI facilitate the measurement of statistics, including higher moments, for both exon and intron variability across a wide range of expression values. We measure differential GINI change between treatment groups by calculating the relative delta GINI change (${\delta }_{GINI}/GINI)$. The statistical basis for significant ${\delta }_{GINI}$ level is explained in the supplementary section S9.

Tests for significance (p-value) were performed according to the negative binomial distribution (NBD). Expression values have been shown to be more consistent with the negative binomial distribution than lognormal or normal distribution^6,8,11,58,60–63^. In particular, modeling the RNA-Seq counts using NBD and linking the variance to the mean of counts, provides the strategy to account for overdispersion in data. Significance levels were adjusted for false discovery by using the Benjamini–Hochberg procedure. A false discovery rate (FDR) of 0.1 that would hold for both exon and intron data translated to a p-value of 0.0011—therefore, a p-value of 0.001 was used (see the supplementary material for further discussion).

Change in correlation p-values were calculated by using Fisher’s formula. We used “exon:intron” throughout this work to refer to the joint (Matched) relationship between exon and intron counts for a gene within and across samples and define the specific relationship in the context of the analysis. For correlation analysis between exon and intron counts, we used the standard Pearson correlation coefficient either with or without transforming the values to log2 scale. We also used Spearman’s rank correlation to evaluate potentially non-linear relationships. Both Pearson correlation and rank correlation measure the degree to which two vectors (exons and intron read counts) are related—independent of the absolute scale of exon and intron values. Pearson correlation was used since there were no noticeable nonlinearities (see supplementary material for a comparison). We used robust regression to establish the overall exon:intron relationship between rescaled exon and intron counts. Rescaling was performed to reduce the impact of heteroscedasticity. It was accomplished by dividing the counts by the respective lowest count value for each gene. Slope analysis was used to rigorously assess the statistical significance of exon and intron changes at the aggregate level. We obtained a robust estimate of the regression slope for the correlation relationship between adjusted relative exon and intron gene expression across samples and evaluated the significance of these differences in the slope of exon/intron correlations. The significance test provided a p-value for difference in slopes^64^.

Cluster analysis was used to identify and visualize the relationship between exon expression values before and after normalization by the corresponding intron counts. This ratio provided per-sample information, and its use was justified because the ratio relationship line had zero intercept. Data for exons were normalized for each gene by dividing all expressions levels with the lowest corresponding expression level. Data for introns were treated similarly. For the exon only clustering, the normalized values were subsequently transformed by log_2_. Exon–intron clustering was performed after taking the ratio of the normalized count values (exon/intron) and performing a log_2_ transformation. To mitigate divide-by-0 in cases where the lowest expression level was equal to zero we added one to all normalized intron values. The Pearson correlation coefficient was used as the distance measure in the K-means algorithm^65^. Clustering results were evaluated by calculating the frequency of co-clustered pairs—referred to as the Adjusted Rand Index (ARI)^49^. An ARI value close to zero indicates that the clustering models were dissimilar, while perfect similarity was indicated by an ARI value of 1. (see additional information in Supplement S4).

The rich content of RNA-Seq data continues to foster the development of several methodologies and algorithms. Each software package provides tunable parameters and comes with assumptions appropriate to the methodology. To carry out our comparison, we have focused on one of the most commonly used workflows in differential gene analysis. Our comparison set of genes is obtained by normalizing for library size, performing a significance test using NBD (to account for overdispersion), correcting for FDR, and selecting for genes with 2 × expression change (see supplementary material for further information). Our extended set of genes is obtained by merely amending the final step and including genes that show distributional or relationship (exon–intron) changes.

## Results

The results of our investigation are organized into three sections. In the first section, we demonstrate that when exons counts are normalized by the corresponding intron counts, the resulting co-expression profile (log_2_ scale) in a control population is markedly different from the profile of exon-only counts. In the second section, we investigate the three quantities, exon:intron correlation, exon GINI, and intron GINI (MEI), and establish that each of these quantities can vary independently. These results demonstrate that relationships between exon and intron counts, and the variability of counts as measure by GINI should be considered to be parameters of interest. In the third section, we use MEI analysis to establish an expanded differential gene expression profile and use it for the selection of an enlarged set of differentially expressed genes. The additional genes identified by employing MEI analysis are associated with functionally significant immune pathway networks that could be contributing to the health status of the population under study. We contrast the expanded set of genes with the significantly smaller set of genes that were not associated with any pathway based on standard measures of statistical significance. In particular, we compare the significance of gene pathways highlighted by the network of genes from MEI results with those obtained from the standard analysis. Based on these observations, we propose that the application of matched exon:intron GINI scores should be considered as a means to further amplify the power of the standard analysis criterion for differential gene expression analysis.

### Marked difference in the co-expression profile of exon-only and exon:intron ratio data

Gene co-expression network analysis has been used extensively to infer gene function and gene–disease associations from genome-wide gene expression^66–75^. The idea is to construct networks of genes with a propensity to co-activate across a group of samples and subsequently probe this network using additional information. For example, differential co-expression analysis can be used to identify genes with varying co-expression partners under different conditions, such as disease states, tissue types and developmental stages. Differential co-expression analysis is premised on the idea that the pattern of co-expressed gene partnerships is more likely to provide information about the regulators that underlie phenotypic differences. Based on the exponential decay model of free fragments, the value of log_2_(exon/intron) can be viewed as an aggregate decay time constant. Therefore, co-expression profile differences exist between exon-only expression data and exon:intron expression data provides important clues about the nature of information provided by each. Clustering, a well-established method for investigating correlation structures, was therefore used here.

For exon-only data, we clustered genes first based on exon log_2_ transformed data. In order to incorporate the intron data for clustering, we used the matched exon:intron ratios on log_2_ scale; this ratio was calculated for each gene and for each sample and used as the clustering variable. Prior to log_2_ transform, we relativized exon expressions by dividing exon counts by the lowest exon expression for the selected gene. An identical computation was performed for introns using the lowest intron count. This procedure created two feature vectors of length 35 for each gene (n = 35)—one feature vector for the exon-only data, and another for the exon:intron data. We used the K-means data clustering technique with Pearson correlation as the distance measure. The number of clusters was automatically determined by a statistical optimization procedure (AIC)^76^. Because we intended to use the public NCBI data set later, we included 3,943 genes from the in-house control data set (81%) that were also found to be in the NCBI “singular count” data set in our analysis (the intersection of the two sets of genes). The clustering algorithm identified three clusters for the exon-only data, and four clusters for the exon/intron data (Fig. 1).

The three-cluster grouping identified for the exon-only data (Fig. 1A) were qualitatively and quantitatively different from the four exon/intron clusters in panel B. In addition to the presence of different number of clusters in panels A and B, significant quantitative differences were observed between the two clustered data sets. To demonstrate quantitative differences, we calculated the frequency of co-clustered gene pairs adjusted for random chance (Adjusted Rand Index or ARI). The calculated ARI of 0.12 indicated that the two clustering models are highly dissimilar (perfect similarity is indicated by ARI = 1 and numbers close to 0 are indications of significant dissimilarity). The color patterns observed in the control data set used red to indicate a positive z-score (> 1) and blue to indicate a negative z-score (< 0). The color patterns could be read in the vertical or horizontal direction similar to a barcode. Each vertical slice (column) represents the barcode profile for one gene across the 35 samples, whereas the horizontal barcode patterns may represent the global regulatory state of the entire genome for one individual. In one interpretation of the barcodes, we considered a lower or higher relative abundance of intron reads as a proxy for the signaling of regulatory events. For example, the blue-red-blue-red pattern is common to several control individuals near the bottom of panel B (horizontal slices) while individuals in the middle of panel B are more likely to follow a blue-blue-red-blue pattern (see also Supplement S4).

In summary, the co-expression profile of the 35 members of the control group identified four unique motifs of exon:intron ratio expression. The observed pattern was readily differentiated from the exon co-expression profile for exon-only data (panel A). These observations demonstrate that the incorporation of intron data (exon:intron ratio) significantly alters the correlation structure of the gene expression data. Additionally, the pattern of exon and intron changes across samples and within one individual are noteworthy; these patterns are discussed further in supplement S4 and Fig. 3s.

### Matched exon:intron relationships and changes in variability of exon or intron counts provide additional information

Observations in the previous section guided our search for additional quantitative measures that could elucidate the regulatory role of introns. We considered changes in the variability of exon expression, intron expression, and the relationship between exon and intron expressions as the most direct candidates for consideration. We used GINI for the measure of variability, and the slope of exon:intron correlation as the measure for the relationship between exon and intron expression levels. We utilized all 4,865 genes identified as having singular counts in the 35 in-house control subjects.

**Figure 2:**
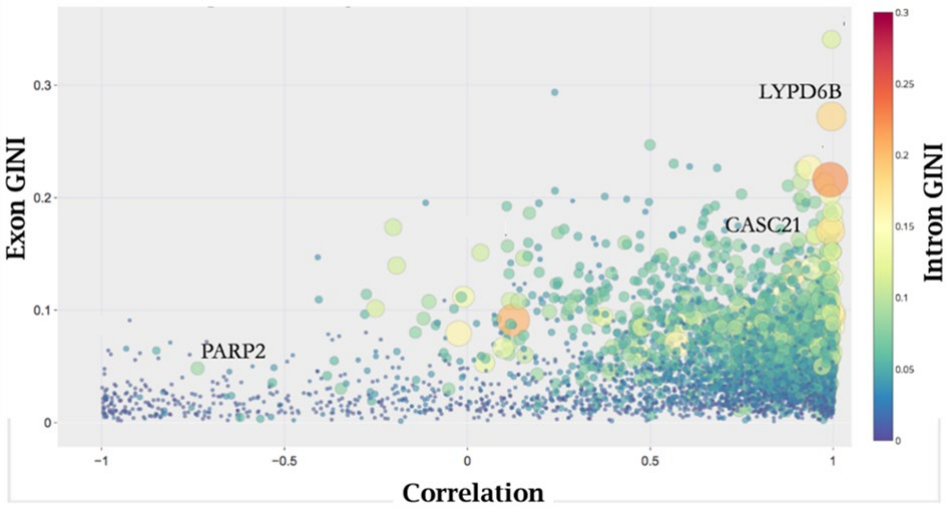
The global relationship between exon and intron read levels. Circles depict quantities derived from matched exon and intron counts for 4,865 genes in a control sample of 35 individuals (n = 35). The x-axis shows the relationship between exon and intron read levels based on the computed Pearson correlation coefficient “r” obtained by robust regression of matched exon and intron counts across the 35 samples. The left y-axis shows exon dispersion as measured by GINI coefficient. The intron GINI coefficient is depicted by the size and color of the circles as defined on the right y-axis (for clarity, both color and size encode the intron dispersion level). Several relationships can be observed. Correlations between exon and intron counts range from highly correlated (1 > r > 0.95) to anti-correlated (− 0.4 > r > − 1). Exon read levels do not provide a consistent report on intron counts; there are numerous genes in the low correlation area, (0.4 > r > − 0.4), where both exon and intron read levels have low dispersion. Three genes exhibiting markedly different correlation and dispersion levels are identified in the figure with a “ + ” symbol.

We revisited the exon/intron ratio relationship described in Fig. 1, by considering the linear correlation relationship between exon and intron counts. Initially we used both the Pearson correlation coefficient and the rank correlation coefficient to ensure that both linear and potentially non-linear relationships were evaluated. Because there was no significant difference in the results, Pearson correlation coefficient analysis was used (Supplementary S5, Fig. 4s). According to standard statistical theory, a low correlation or anti-correlation relationship between exon and intron expression levels would indicate that simultaneous use of both intron and exon data could provide information beyond either exon or intron data alone. The control results demonstrated that more than 1/3 of the genes surveyed had low correlation values (r < 0.5), thus the expression level of one (exon or intron) could not be accurately inferred from the other (Fig. 2).

Next, we compared the variability of exons and introns, for each of the 4,865 genes in the control data set, by calculating the GINI coefficient for exon and intron expression levels. We created two lists by rank ordering genes according to exon variability (low to high) and by intron variability (low to high). The two lists had significantly different rank ordering of genes. For example, among the first 500 genes in both lists only 250 genes were common to both lists; this corresponds to an ARI value ≤ 0.3 (see Supplement S6). The absence of rank order correspondence further supported our view that exons and introns had significantly different expression variability profiles.

The simultaneous consideration of exon:intron correlations, exon variability, and intron variability, is illustrated in Fig. 2. The figure summarizes and visually confirms that the global profile of gene expression data exhibited a range of complex interrelationships. In particular, some parameters showed correlations, while other parameters were uncorrelated, indicating that all three measure should be considered. For example, although LYPD6B and CASC21 genes showed highly correlated exon and intron relationships, the LYPD6B gene showed high exon and intron variability while the CASC21 gene exhibited lower exon variability and moderate intron variability (Fig. 2). In contrast, PARP2 exhibited low intron and exon variability, but its exon and intron expression were effectively anti-correlated. Changes in these three parameters may be indicative of different regulatory events, and all three parameters were considered during differential gene expression analysis.

Summarizing the results so far, we note that co-expression profiles for exon:intron ratios were shown to be distinctly different from the profile for exons alone (Fig. 1), thus strengthening the hypothesis that the exon:intron pairs present new information as compared to exon expression values alone. Moreover, computational experiments suggest that the formation of the four tightly related groups in the exon:intron data was not a fixed parameter resulting from random gene degradation alone (see Supplement S9). We also noted that each of the three parameters presented in Fig. 2 may carry independent information. Next, we determined that all three measures provided independent information useful for MEI analysis.

### MEI analysis identifies additional differentially expressed genes

We used MEI to further examine differential global changes in gene expression in an archived NCBI data set (Control (n = 12) vs. polyarthritic Case data (46)). MEI analysis was performed on 4,294 genes for which the data contained singular counts (see Methods section). As noted previously, approximately 90% of these genes were also identified in our in-house controls despite differences in the method of RNA extraction and sample preparation. A commonly used analytical approach is to select a threshold p-value for exon expression changes (E_p_), and additionally require a twofold change in the mean expression change (E_m_). For the MEI analysis of the Control and Case NCBI data, in addition to E_p_ and E_m_ (Table 1), we used the following MEI measures in the differential analysis: (1) intron p-value (I_p_), (2) the p-value for exon:intron correlation coefficients change between the two data sets (C_d_), , and (3) relative exon GINI change (G_e_), and (4) relative intron GINI change (G_i_). Changes in correlation between matched exon:intron counts were used as a more rigorous proxy for comparing the clustering patterns of genes.

**Table 1:** Parameters for differential expression analysis. The parameters considered are: p-value for exon expression change (E_p_), mean-fold change (E_m_), p-value for intron changes (I_p_), p-value for correlation changes (C_d_), and exon and intron differential GINI changes (G_e_ and G_i_). The combination of Ep and Em (first two rows), is commonly used for the detection of expression change. Our analysis expands the parameters to include the combination of E_p_ with one of E_m_, I_p_, C_d_, G_e_, G_i_.

Differential measures	Symbol
Exon p-value	E_p_
Mean-fold change	E_m_
Intron p-value	I_p_
Correlation differential	C_d_
Exon GINI differential	G_e_
Intron GINI differential	G_i_

The following thresholds were used to establish a baseline for identifying genes that exhibit significant change between the two groups. For the standard statistical criterion, we used E_p_ < 0.001 p-value as the significance level (negative binomial test), which yielded a set of 346 genes. In the identified data set of 346 genes, 25 genes were found to have at least a twofold change. The set of 25 genes with E_p_ < 0.001, and twofold expression change did not identify a significant enrichment for gene ontologies based on the String-db enrichment analysis^77^. Two genes, ARG1 and TBX1, involved in negative regulation of T-helper 2 cell cytokine production, with a calculated false discovery rate (FDR) = 0.0172 (GO:200052) were identified (see Supplement S7). The choice of p-value < 0.001 as the more stringent criteria was used throughout our analysis to guard against false positive selections due to numerical computation differences, or assumptions related to the underlying distribution of data^78^.

In order to explore additional prospective genes, we used MEI calculations and applied the following quantitative criteria for selection. We required a minimum of two significant differential changes as a criterion for inclusion in the list of genes of interest. For criterion 1, as in the standard analysis, we required that all genes satisfy a differential exon expression change (control vs. case) at the significance level of E_p_ < 0.001 (NBD). As noted above, the application of E_p_ < 0.001 yielded 346 genes. For the second criterion, we required that all candidate genes satisfy one of the following criteria: intron p-value significance of I_p_ < 0.001 (NBD), more than twofold change in either exon or intron GINI value between case over control, or significant correlation differential value of C_d_ < 0.005 (Fisher test). The designated GINI change threshold was selected to identify genes that displayed a significant change in variability between control and case populations. The Fisher test threshold was set consistent with the strictness criterion for testing a change in correlation.

**Figure 3:**
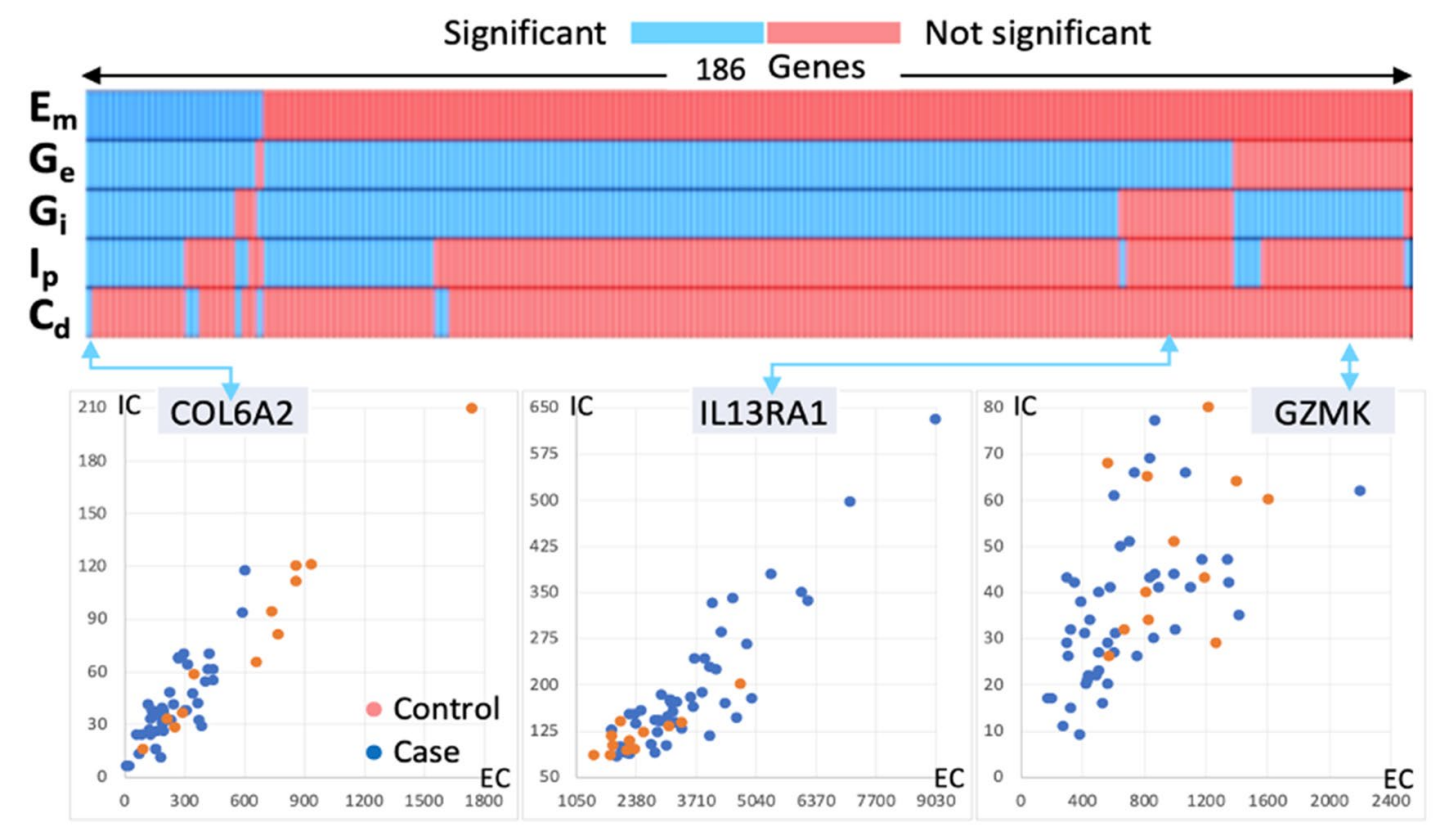
Contribution to the expanded gene list and example gene profiles. The heatmap identifies each parameter with significant change (Table 1) between control (n = 12) and case (n = 46) for each of the 186 genes (Ep < 0.001). The map is organized by sorting each row in priority order (top to bottom). Em is based on the standard twofold change value. Scatter plot of three genes corresponding to specific parameter change conditions is linked with an arrow to their location on the heatmap. For example, COL6A2 is the first entry and it has significant changes for all 5 parameters, while IL13RA1 has a significant change for G_e_ only (exon GINI). Note that significant changes in G_e_ or G_i_ (2 × change corresponding to p < 0.001) account for all selected genes except FAM118A (last column). In the case of FAM18A, the significant differential change is in intron expression. The mnemonic EC stands for exon counts (x-axis; IC stands for intron counts (y-axis).

The addition of the complementary selection criteria reduced the list of 346 genes to 186 genes (slightly more than sevenfold increase in the number of gene candidates as compared to the standard method). The contribution of each criterion to the selection criteria is shown in Fig. 3. It is important to note that the list 186 genes identified with our expanded selection criterion were simultaneously detected by 1–5 of these parameters thereby indicating that complex coordinated changes appear to be occurring among these genes. We examined several genes from this list and plotted the exon:intron relationship in Case and Control populations for three specific genes (Fig. 3). These prototypical genes elucidated in more detail the changes in exon:intron relationships between the Control (blue circles) and Case (red circles) (Fig. 3, COL6A2, IL13RA1, and GZMK genes).

The gene COL6A2, which has been associated with early onset arthritis is the single gene that exhibits significant changes for all of the measures proposed in Table 1. In addition to the reduced range of expression observed in the figure, both exons and introns exhibit reduced variability (G_e_ and G_i_) as shown (Fig. 3, COL6A2). ARG1 has a scatter profile similar to COL6A2 (not shown). It is an anti-inflammatory gene with associations to arthritis^79^, and exhibits changes in all measures except C_d_. It is interesting to note that all genes differentially identified based on the additional criterion of twofold change can also be identified independently by using one or more of the three other criteria identified in Table 1.

While the expression level changes for IL13RA1 do not meet the twofold threshold, GINI calculations show significant changes in exon variability. Visual inspection of the scatter plot for this gene suggest differential changes (Fig. 3, IL13A1). Interestingly, this gene has been implicated as a circulating biomarker for arthritis^79^. GZMK, a serine-protease member of cytotoxic lymphocytes capable of recognizing, binding, and subsequently lysing target cells, has been shown to play a role in inflammatory response^79^. While GZMK has a statistically significant differential change between case and control, it does not meet the twofold criteria; however, intron GINI differential change of larger than twofold supports the inclusion of this gene. RNF11 gene is ring finger protein^79^ with a scatter plot profile (not shown) similar to GZMK. It is involved in the modulation of inflammatory signaling pathways. Similar to GZMK, the fold change calculations do not reach the twofold threshold, but in this case the exon GINI score changes by more than twofold between control and case samples. Another example where twofold change threshold is not reached is the NCR1 gene. The data exhibits significant intron and exon changes (p < 0.001), while correlation between exon and intron values is maintained in both the control and the case groups (figure not shown). NCR1 is a cytotoxicity activating receptor that may contribute to the efficiency of natural killer cells^79^.

Additional criterion for identifying genes of interest was based on significant changes in exon:intron correlation (C_d_ < 0.005 using Fisher’s test). OAS1, a protein that is known for antiviral and possibly apoptotic activity^76^ provides an example for differential change in correlation profiles (figure not shown) between the Control and Case samples. The control data for matched exon and intron values is correlated (r = 0.89), the case data exhibits a loss of correlation (r = 0.33). However, for the NCBI data set examined here, all genes with significant correlation structure changes can also be selected by using either exon GINI or intron GINI changes. This behavior may be unique to this data set because our earlier observations indicated that exon and intron GINI and correlations were not always linked (Fig. 2). Therefore, it may be useful to retain C_d_ as a selection criterion for further evaluation.

**Figure 4:**
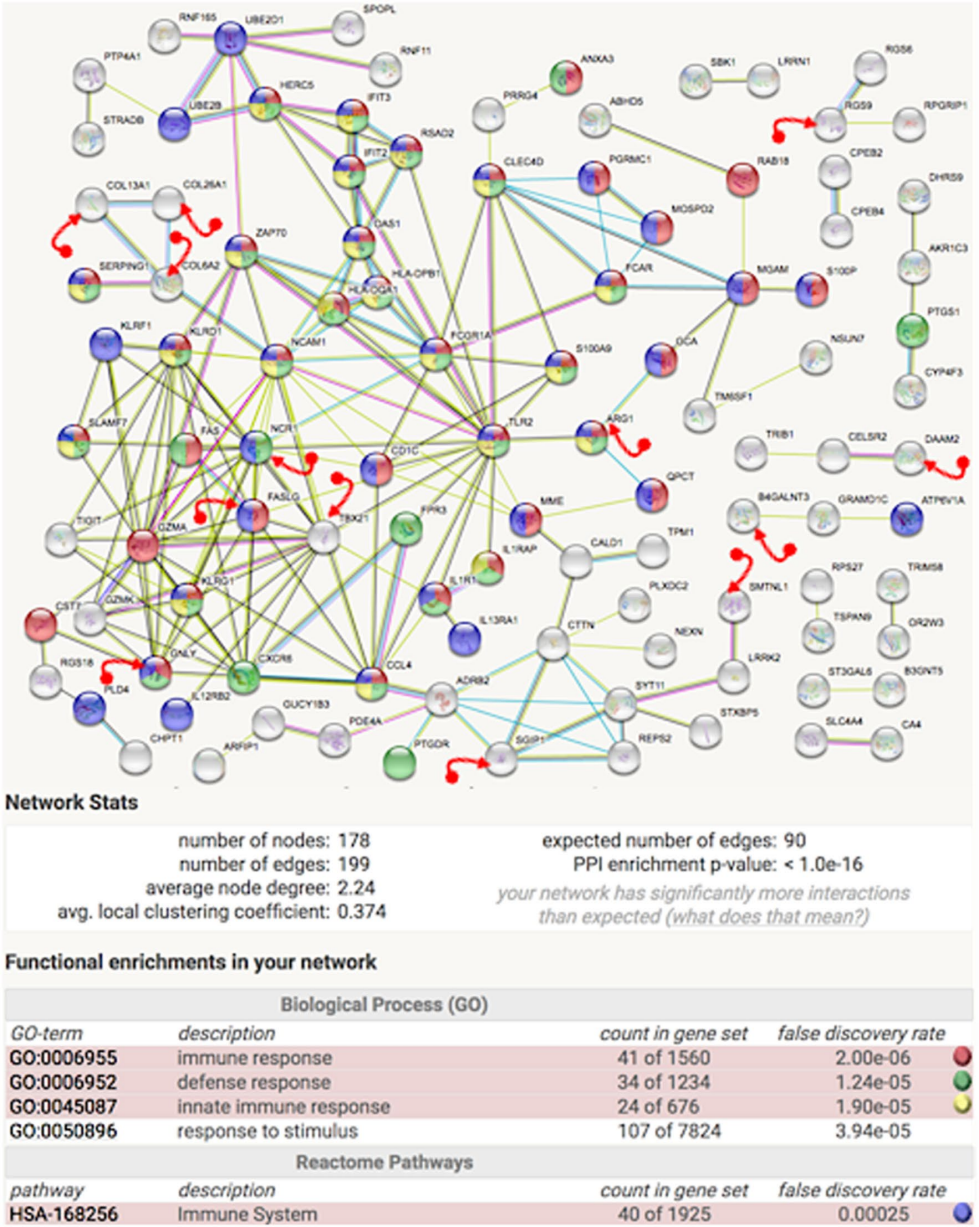
MEI expression analysis identifies significant immune-related interactions . Image from String db^77^ analysis of the 186 genes candidates highlights a significant immune related network involving 178 annotated genes. To increase clarity, isolated genes not showing associations with other proteins were not included. The 13 genes marked with the red arrow symbol are genes that were identified in the smaller set of 25 genes based on the standard analysis (see Supplement S7). The enrichment results highlight immunity, which is consistent with the expected differential activation in the polyarthritic case vs. control population in the study. The legend in the figure provides the color codes identifying the biological processes and the associated pathways.

Differential changes in the identified parameters distinguished 186 individual genes relevant to polyarthritic condition in the study. The list included the initial list of 25 genes identified earlier by the standard approach. More importantly, String-db analysis^76^ of the expanded list of genes revealed a significantly enriched interaction (PPI enrichment < 1E-16) that identified key immune-related networks (Fig. 4). The addition of approximately 150 new gene candidates resulted in a significantly enriched network which placed the smaller set of 25 genes in the larger context of the immune network. For example, associations between ARG1 and TXB25 were completed to form a circuitry. The immune response, defense response, and innate immune response pathways (Biological Process GO) identified by enrichment analysis are congruent with the expected involvement of an immune interrelationship in the polyarthritic group. Moreover, the enrichment of an additional 178 annotated genes was not caused by an “immune enrichment bias”, because the complete set of 4,294 genes was not enriched for immune response pathways according to Strings-db^77^ and Panther db^78^.

## Discussion

In this report we introduced MEI analysis as a complementary approach for the study of exon and intron relationships and the identification of differential changes in the gene expression circuitry. Our proposed approach to differential analysis contributes to the ongoing research on differential measures by expanding the opportunity to identify gene targets of interest. Our consideration of changes in expression variability and the relationship between exon and intron counts build on previous observations. Variability of expression in exon levels, considered to be important in certain conditions^50^, has been incorporated into MEI by using the GINI coefficients. Intronic RNA reads are a significant fraction of data from RNA-Seq experiments, and we have shown that the application of MEI analysis to standard RNA sequencing results and the use of GINI coefficients provides a new approach for analyzing differential changes. We identified the additional patterns of exon:intron correlation and its changes as a complementary measure for differential analysis. Although exon:inton correlation changes did not identify genes that had not been identified by the other proposed measures (Fig. 3), we noted many anticorrelated genes with changes at a lower threshold (Fig. 2). Therefore, we consider this measure to be a valuable adjunct that may depict more dynamic changes in future studies.

MEI analysis captures shifts in gene co-expression patterns that may contain information relating to regulatory events. These shifts are difficult to discern through the use of standard expression analysis because changes to individual exon expression levels may be comparatively small when measured against the landscape of variability occurring across multiple genes. In addition, exon–intron expression is affected by time-dependent processes and further clarification of MEI results may be possible by studying these changes over time. The characterization of additional modes of analysis highlight the fundamental importance of devoting future studies to determine the extent to which changes in the pattern of intron expression may contribute to the modulation of physiological processes. For example, MEI changes in G_i_ without changes in G_e_ suggests a change in the distribution of values of introns but not exons. For genes with significant change in E_p_ (the genes we consider), this result reflects changes in the relationship between exon and intron levels—for example, the emergence or disruption of correlations between exon and intron levels. The combined impact would suggest a regulatory change in the role of introns.

A less strict cutoff value for E_p_ and E_m_ provides an alternative for recovering more differentially expressed genes. However, a less strict E_p_ or E_m_ will also yield many genes that are potentially false positives (increase in the FDR). For example, in order to recover all MEI significant genes by relaxing the E_p_ or E_m_ criteria, we had to consider a large number of additional genes (a minimum of ~ 385 additional genes). Since there were a total of 373 genes at our FDR threshold, many of the additional genes are likely false positives. A more comprehensive assessment of blood transcript levels with tight computational controls over model assumptions, and the use of both intron and exon expressions, may improve the clinical usefulness of the data and the predictive value of these studies in various disease states. The complementary strategy outlined here presents new opportunities for analysis of the massive trove of data in existing databases.

In addition to its potential to provide complementary information in differential expression analysis, MEI analysis may be useful for other applications. For example, select genes that are known to fall within a defined range of exon–intron correlations, may be useful for identifying cases for further within-group validation, quality checking, or detection of technical issues with sample and/or data processing. Moreover, additional MEI analysis of public data sets may make it possible to construct a careful and detailed catalog of intronic region decay rates for specific genes in specific tissues. Such a study would require a great deal of computational resources, but the resulting catalog of data may be highly informative in the interpretation of new experiments in the same tissues.

The richness and complexity of RNA-Seq data has led to the development of a host of software tools and computational pipelines. In turn, new software tools with additional internal validation criterion may help set a gold standard to evaluate and compare sequencing outcomes when different pipeline employed by laboratories arrive at disparate results. As an additional source of information, MEI analysis may also motivate the development of richer software tools for RNA-Seq analysis.

Our use of “singular”, high quality counts places more emphasis on the genes that yield counts that are not multimapped; only ~ 50% of the total reported genes register unique exon and intron counts. Using high-quality reads which are effectively unique as the basis of our expression study has the distinct advantage of simplicity—in essence, the count assignments are model-free. Because model-free counts are likely to be more stable and computationally reproducible, they provide more robust results and can be used as an initial probe of biological events. In this context, the cutoff criteria for filtering matches (MAPQ) can be relaxed in order to capture more genes. The use of model-free counts can then be followed by incorporating model-based algorithms with greater coverage to extend the information across the genome. Additionally, genes without intron counts can also be included. The initial application of a more stringent analysis of RNA sequencing data may provide the basis for a more comprehensive analysis of the complete gene pool and would require the development of additional software.

The reduction of technical noise and variability at the sample processing stage can further aid in curtailing information loss. Many RNA sequencing processing pipelines routinely employ data transforms to mitigate noise and variability, but the application of these transforms may be counterproductive if this variability contains important biological information. ERCC spike-in controls can provide reliable data for sample-to-sample calibration by referencing to a proportional (or relative) value, as done here, as opposed to referencing to an absolute concentration. Efforts to improve the precision of read quantification and reduce sources of experimental variability will be pivotal in identifying tightly regulated gene expression patterns within a group of genes. For example, if genes within a regulatory cluster begin to display large changes in exon:intron expression, these changes may identify altered states of gene regulation as previously illustrated by the genes identified in Figs. 3 and 4.

The investigation into introns and intron variability was motivated by our interest to further understand the relationship between exon and intron counts and its role in elucidating regulation. Although existing approaches to differential expression are important, expanding these methods in order to include intron expression as well as exon and intron variability enriches the analysis by considering the potential regulatory information in RNA-Seq data. For example, the consideration of differential changes in the MEI measures expanded the initial group of 25 genes to a more comprehensive list of 186 genes that play a prominent role in the immune circuitry relevant to the condition under study. While the MEI approach may pose a small additional computational burden, it has the potential to provide significant insight into potentially important regulatory pathways. In turn, an improved understanding of the global nature of these regulatory changes may contribute to alternative treatment strategies and potential new arenas for pharmaceutical intervention as well as personalized medical treatments.

## Supplementary information

Supplementary information
