# Supplementary material for: Coordinated analysis of exon and intron data reveals novel differential gene expression changes
Source: Sci Rep. 2020 Sep 24;10:15669. doi: 10.1038/s41598-020-72482-w (PMC7515875; doi:10.1038/s41598-020-72482-w)
Supplement: Supplementary file 1 — Supplementary information [file 41598_2020_72482_MOESM1_ESM.pdf]

Coordinated Analysis of Exon and Intron Data Reveals Novel Differential Gene Expression Changes

Hamid R. Eghbalnia[*1,2], William W. Wilfinger*[3], Karol Mackey*[3], Piotr Chomczynski*[3]
(1) University of Wisconsin-Madison, (2) University of Cincinnati, (3) Molecular Research Inc.
** Corresponding author*

Overview

The supplemental material in this document provides additional supporting data and more detailed explanations of the studies reported in our manuscript and it is organized into nine sections designated S1 through S9. The main text of the manuscript references this content by using the section designations; for example, "Supplement S1". The ancillary results presented in S9 provide supporting documentation for the analysis employed in the body of the manuscript.

Section S1. Samples and Workflow

Samples.

The material related to *negative control* and *workflow* is provided for completeness in order to support the main manuscript. **Negative control PCR experiments.** qPCR substantiates the expression of intron counts and confirms that intron counts are not due to DNA contamination. Furthermore, rank correlation of unique exon expression levels with qPCR values, combined with correlations observed in ERCC levels, provides strong support for the cogency of using unique expression values. Absence of gDNA contamination was verified through negative control PCR experiments (the omission of reverse transcriptase in RNA samples during cDNA preparation did not yield amplification product after 50 cycles of PCR). In order to further verify the presence of intronic counts, a subset of genes was selected, the RNA was reverse transcribed and intron sequences were identified and quantified by PCR.

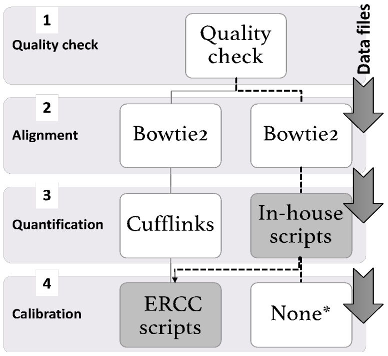

Figure 1s - ***Workflow.*** *Unique exon and intron values were obtained in four steps. After quality control (step 1), raw exon and intron values were generated by using standard tools (Step 2). Unique counts, and statistically adjusted counts (Cufflinks) were extracted in (Step 3). Unique counts used in-house scripts. If ERCC data was available, the data were calibrated (step 4), otherwise unique count data was adjusted to total counts.*

Workflow. Raw data from 35 samples in the FASTQ format was submitted for quality control in order to evaluate adapter contamination, average base quality score per read, the GC content distribution, and other relevant parameters. The software FastQC was used to perform quality control. All samples received passing marks according to FASTQC benchmark criteria. FastQC software was further utilized to verify the quality of aligned BAM files. The alignments were generated by the Bowtie2 aligner, a BWT-based unspliced aligner, with the recent addition of supported gapped alignments. Bowtie2 extracts

multiple substrings or seeds from each read and aligns them using a BWT approach with no gaps, then extends alignments using a Smith-Waterman-like scoring scheme. The reference genome GRCh37.p13 [hg19] was augmented with data for ERCC spike ins (provided by the manufacturer) in order to obtain ERCC spike in counts. We used the results from Cufflinks for comparative analysis with the unique counts we extracted using HT-Seq and our in-house scripts.

Figures generated in this project used one of the following software packages: Python plotting functions using Bokeh, Matlab plotting functions, Excel plotting functions, and Strings-db plotting functions. No permission or citation is required for these packages. However, we acknowledge the use of Python (https://www.python.org), Bokeh (https://bokeh.org), Matlab (https://www.mathworks.com), Strings-db (https://strings-db.org), and Excel (https://www.microsoft.com) in generating figures.

Section S2. Singular counts

A crucial step in quantifying RNA-seq data is mapping sequence fragments, the reads, to the reference sequence (genome or transcriptome). Because reads are typically short fragments and there are base errors, it is not always possible to get a single best match. Generally, there are other challenges including paralogous gene families, repetitive sequences, and the high sequence similarity between alternative spliced isoforms from the same gene that will cause mapping ambiguities. In short, these factors will result in the assignment of some reads to more than one mapping location – sometimes these are called multi mapped reads. These factors impact data analysis, and addressing these read mapping difficulties is crucial for differential expression studies. We have referenced some software tools that have been proposed to handle multi-mapped reads such as allocating them in proportion to the number of uniquely mapped reads, or using a generative statistical model and associated inference methods to address the computational issue of read mapping uncertainty. A common approach is to use only those reads with a "single best match", and this is the approach we have used. Our use of what constitutes a single best match is stated in the manuscript. To arrive at this list, we used the HT-Seq software in the "unique count" mode (described further below). We also used a set of custom-made scripts that extracted the unique counts from SAM files for each sample (also see below). We then correlated the unique counts obtained from our scripts, HT-Seq, and the values obtained from Cufflinks for the purpose of validation. For our "singular counts" these values were correlated (r> 0.97) because our selected genes had a very small fraction of non-uniquely mapped data (generally less than 2%).

Bowtie2 output is in SAM format, and the format is specified in the SAM format specifications document. The data contains 11 fixed fields and additional fields are optional. For our purposes, the important fields that are used are Fields 5 (MAPQ), 6 (CIGAR), and Fields 12+. For example, a CIGAR string could be as follows: 50M300N50M. This would indicate that 50 bp of the read matches to a region (50M) and then there is a 300 bp skip (300N) and then the last 50 bp of the read match the sequence (50M). Additional available fields that we read are:

```
Fields 12+:
AS:i:<N> alignment score
```

```
XS:i:<N> alignment score for next best alignment (only reported if >1 alignment found)
YS:i:<N> alignment score for mate (PE only)
XN:i:<N> number of ambiguous bases in the reference covering the alignment (#Ns)
XM:i:<N> number of mismatches in alignment
XO:i:<N> number of gap opens
XG:i:<N> number of gap extensions
NM:i:<N> the edit distance
MD:Z:<S> A string representation of the mismatched reference bases in the alignment.
```

We use the information in the fields above to select singular counts. In order to access this data and generate a table, we use a number of utilities available as part of the `samtools` functions. For example, by using the following:

```
samtools view -h my_R1.bam | awk '$0 ~ /^@/ || $5 ~ /N/' | samtools view -b > f.bam
```

we convert the bam file `R1.bam` to sam format, and we include the header (-h). The awk command (part of Linux utilities) filters the reads that start with @ (headers), or lines that contain N in the 5th column (the CIGAR string). The second view statement converts back to bam format and writes to a file called `f.bam` which contains a list of all annotated *and* unannotated splices. We take into account differences in the reporting of MAPQ score. The maximum MAPQ value that Bowtie 2 generates is 42. In contrast, for example, the maximum MAPQ value that BWA will generate is 37. Two other pieces of information are used (when available) in order to perform additional quality assurance. The CIGAR string is used to additionally check for the number of aligned bases, and the optional 12+ fields are considered; specifically, for AS and XS information. All AS and XS were as expected for 0-5 mismatches: 0, -6, -12, -18, -24, and -30 respectively. The MAPQ for read1 was 42 because there was only a single perfect alignment. Our evaluation of the previously described fields shows that a MAPQs of 40 indicates one mismatch. We use the information in the CIGAR field to check that the match is the results of a contiguous read of bases with no gaps. In principal, it is possible to exclude multireads from the data set by using MAPQ > 2 which would also exclude any uniquely mapping reads with >=4 mismatches over high quality reads of bases. However, if we assume an average quality of bases, then a higher MAPQ would be needed.

Our protocol for obtaining intron read data follows a similar approach, but it is tailored to address some unique challenges of introns. There are alternative ways for counting introns, and we have investigated several approaches. For example, one approach is to analyze all intronic intervals present in at least one annotated transcript model. While this allows to screen the largest set of candidates, it introduces the additional complexities of properly defining the peaks of intronic alignments caused by expressed alternative exons and redundant IR calls due to overlapping introns. We use the following approach which count *introns* that do not overlap with any annotated exon. They are obtained by subtracting merged exons from genes. This approach simplifies the analysis of introns flanked by exons with known alternative donor sites, and ignores introns fully overlapped by annotated exons.

In addition to the use of our own counting method, we used utilities in the software `bedmap` to map reads to introns, using:

1) the `--count` operator to perform counting of reads overlapping by our criteria; and,

2) the `--indicator` operator for a logical indication of reads contained entirely within the intron.

For instance, to count reads that overlap introns by at least 45 bases, we use `--count` to count and `--bp-over 48` to specify the minimum overlap threshold:

```
$ bedmap --echo --count --bp-ovr 48 --delim '\t' int.bed count.bed > out1.bed
```

To capture introns where reads are contained entirely within the intron, we used `--indicator` to get introns that meet the criteria of full containment of reads, via `--fraction-map 1`:

```
$ bedmap --indicator --echo --fraction-map 1 --delim '\t' int.bed count.bed |
awk '($1==1)' | cut -f2- > out2.bed
```

Comparison of outputs with `bedmap --indicator --exact` revealed where there are commonalities (true/1) and differences (false/0). These operations were performed after a conversion, because our read counts are in the BAM format. We used `bam2bed` to convert the files first, by using: `bam2bed < reads.bam > reads.bed`

Section S3 . Statistical and normalization procedures.

ERCC controls. Although RNA-seq studies have been shown to provide insight into difference in transcription processes, the simultaneous presence of multiple sources of variability can impact the rigor and reproducibility of analysis. Technical control metrics that can be applied to the raw counts can help to improve the reproducibility and validity of differential expression experimental results. External spike-in control ratio measurements can serve as one “ground truth” for benchmarking the accuracy of endogenous transcript measurements. A library of 96 external RNA spike-in controls, developed by the External RNA Controls Consortium (ERCC), is designed to act as the basis for technology-independent control for differential expression experiments. Because normalization factors are computed by comparing expression levels against spiked concentrations, care must be taken to examine the ERCC results for bias as batch effects, pipetting errors, dilution differences, and ratio bias effects have been observed. Since ERCC concentrations are pre-determined by the design of the library, the measured ERCC counts are the determining factor in creating the regression curve, and they have been the basis for the normalization. Therefore, consistency of ERCC values across samples plays a key role in providing a meaningful metric for normalization.

In order to evaluate the reproducibility of ERCC values, we compared ERCC reads for 12 pairs of ERCC library elements with the highest concentration. The comparison was performed by calculating the ratio between the pairs and comparing this ratio to the ratio of concentrations specified by the library. This comparison was performed in two ways – one way was to perform the comparison with ERCC read counts adjusted for the length of ERCC library element, and the

other was to use the read values without adjustments for length. For example, we would expect that two ERCC library elements with equal concentrations (as specified by the library) would record ERCC read values with a ratio of one – because the ratio of their concentrations is equal to one. In all cases, correcting ERCC read values by length improved the ratios – length-adjusted read ratios were closer to the reported ratio of the ERCC concentrations. However, unadjusted ERCC read ratios were always divergent with respect to concentration ratios. For example, the ratios for 18 samples (for the female subpopulation) had ERCC read ratios between 1.25 and 3 for ERCC2 and ERCC74. These values did not compare favorably to the ideal value of one, which is the ratio of concentrations between ERCC2 and ERCC74 as specified in the library (Figure 2s, blue bars with legend "unadjusted"). Linear regression of the ERCC counts vs the known spike-in ERCC concentrations for the purpose of adjusting gene counts causes a further distortion of the internal ratios because: a) the data for the counts is not normally distributed (as reported by the K-W test at 95% confidence), and b) the regression line parameters are coupled through the internal ratios. The non-normality of the ERCC count distribution suggests that the regression solution may be compromised by coupling to the internal ratios. The deviation of the ratios away from their true values may result in a suboptimal solution that is further degraded by the large deviation of ratios from their true values. Our approach to calibration based on these standards is to create a regression curve that utilizes the known ERCC concentration ratios. We now outline this approach.

Our approach uses the ERCC read data as a means of obtaining relative correction values. Variations in ERCC counts for the same spike-ins are assumed to be related to technical differences because the initial concentration for ERCC spike-ins is the same (by design) in all samples. We use this fact to derive a normalization factor for gene expression data. Any sample can be selected as the "base sample" for ratio calibration. In practice, it is best to use a sample that has ERCC expression values close to the mean or median for all the standards in the group. For the sake of clarity, we explain the approach using sample 1 as the base sample. Samples are named using a subscript to indicate their number – for example, the set of ERCC read values for sample 1 is denoted as E_{1j} , and the set of values for sample i is denoted as E_{ij}. The index j in the notation indicates the read count value of j-th ERCC value within the sample (each ERCC spike-in is comprised of several spikes at different concentrations). In order to track the entries and the associated constraints, an ERCC ratio matrix is constructed in the form of a block matrix K. K is composed of three blocks: 1) X connoting sample number, 2) Y the ERCC concentration ratio and , 3) Z the different ERCC ratio pairs. Our next task is to determine an adjustment factor for all samples (sample 1 has adjustment factor equal to 1 by our design. The adjustment is performed in two steps. First, the matrix is factorized into the product of a set of base values and a matrix of weighting factors. If no correction would have been necessary, then the matrix of weight factors would be a matrix containing all 1's. The goal is to adjust the values of the weighting matrix so that all entries are as close to one as possible. We use the known ERCC concentration ratios as the starting set of values to describe the relationship between the columns of the weight matrix. We adjust the weight matrix iteratively based on ERCC spike-ins to obtain the desired adjustments. The results after calibration produce significant improvements as measured by the average weighted distance to their ideal ratios. We illustrate the initial ratios and the results of adjustment for ERCC 74 and ERCC 2 in Figure 2s (orange bars with legend "adjusted").

Note that while the figure illustrates the results for two ERCCs only, the calibration process accounts for all ratios simultaneously and all calibrated ratios are "closer" to the ideal ratio of 1. In this approach, "closer" is measured by using the average deviation across all ratios.

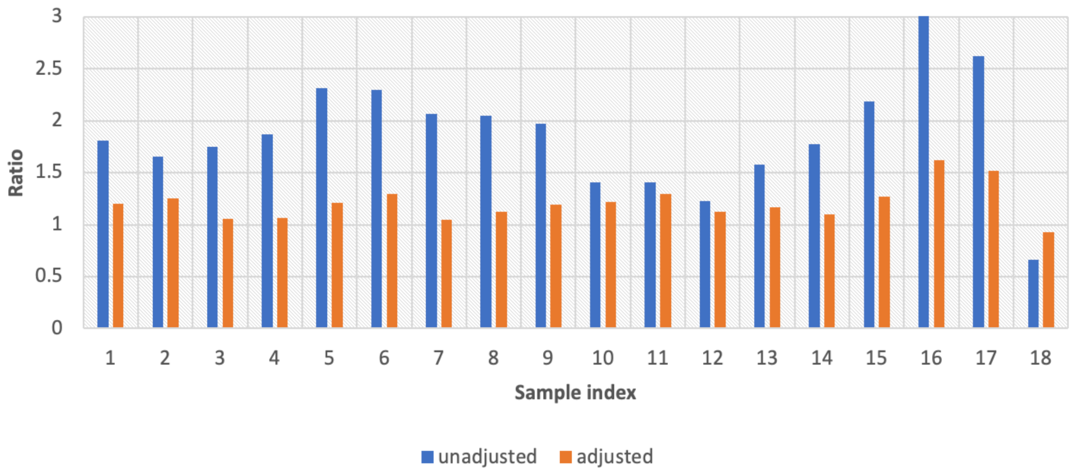

Figure 2s. **Ratio of concentrations reported by RNA-Seq counts.** The figure illustrates the case for two ERCCs; e_i = ERCC2 and e_j = ERCC74 in 18 samples which were spiked with one set of ERCC spike ins (Y-axis is using log_2 scale). The counts are normalized for the different lengths of ERCC standards and the normalized ratios are plotted for each sample (blue bars). The ratios should be consistent with the concentration ratios of ERCC-00002 to ERCC-00074 as reported by the standard provided by the manufacturer which is equal to 1. Note that initially, the closest value to 1 is for sample 12 (~1.25, blue bar), and the farthest is sample 16 (~3.0). After proportional adjustment (orange bars), all of the sample ERCC ratios improve. Two samples (16 and 17) have significantly improved ratios with moderate improvements among the other samples. This may be attributable to their largely skewed initial ratio.

Size factor normalization. Normalization of RNA-seq read counts is an essential procedure that corrects for non-biological variation and other technical issues. In our study, the computed value of an effective library size, referred to as size factor, is used for each sample. By dividing each sample's counts by the corresponding size factors, we bring all the count values to a common scale, making them comparable. Intuitively, if sample A is sequenced N times deeper than sample B, the read counts of non-differentially expressed genes are expected to be on average N times higher in sample A than in sample B, even if there is no difference in expression. We compute size factors using estimation using median-to-ratio method (used in DESeq). To estimate the size factors, take the median of the ratios of observed counts to those of a pseudo-reference sample, whose counts can be obtained by considering the geometric mean of each gene across all samples [3]. Then, to transform the observed counts to a common scale, divide the observed counts in each sample by the corresponding size factor. During size factor computation, we detect and remove outliers from size factor calculation by using the robust statistic $(|x_i| - m)/\overline{m}$, where m is the median of the set of values x_i and $\overline{m}$ is the maximum absolute deviation of values x_i from the median. If ERCC spike-in data are available, we compute the calibration factors as described earlier and compare the results from size factor normalization and ERCC spike-in calibration factor for each sample as a quality check. In our in-house data set, the size factor and calibration factors had ratios ranging from 0.98 to 1.0 (adjusted for total counts). Note that a ratio of 1.0 indicates perfect agreement. All downstream analyses use the size factor normalized values.

The normalization was performed by using the Matlab command lines:

```
count(count==0)=nan;
scf=nanmedian(count./nangeomean(count,2));
count=count./scf;
count(isnan(count))=0;
```

This approach is most appropriate for gene count comparisons between samples and for DE analysis; It is not recommended for within sample comparisons.

While the negative binomial is versatile in having a mean and dispersion parameter, extreme counts in individual samples might not fit well to the negative binomial. For this reason, we perform automatic detection of count outliers. Outlier removal was performed by using the command: `B = rmoutliers(count,'median') ;`
Outliers are defined as elements more than three scaled MAD from the median. The scaled MAD is defined as c*median(abs(A-median(A))), where c=-1/(sqrt(2)*erfcinv(3/2)).

Normalization for dispersion and differential expression. Another important aspect of statistical inference in RNA-Seq data is the differential expression analysis, which is to used to perform statistical tests on each gene to ascertain whether it is differentially expressed or not. Determining whether gene expression in two conditions is statistically different consists of rejecting the null hypothesis that the two data samples come from distributions with equal means. The negative binomial regression model (used in DESeq2) is used in our study. This approach assumes the read counts can be modeled by considering the variance as the sum of the noise term (i.e. mean) and a locally regressed non-parametric smooth function of the mean. Computation was performed by using the function `nbintest` in the Matlab statistical package. The command `nbintest` takes as input arguments read counts for case and control (`cnts(:,case),cnts(:,control)`), and options (`'VarianceLink','LocalRegression'`) that specify the model is negative binomial:

```
nbintest(cnts(:,case),cnts(:,control),'VarianceLink','LocalRegression');
```

The output of `nbintest` includes a vector of P-values – the probability that a change in expression as strong as the one observed (or even stronger) would occur under the null hypothesis, i.e. the conditions have no effect on gene expression. We noted that in our histogram of the P-values there is an enrichment of low values (due to differentially expressed genes).

Multiple testing problem. Because we perform a large number of simultaneous tests on many genes, there is a probability of getting a significant result simply due to chance. In order to account for multiple testing, it is necessary to perform a correction (or adjustment) of the p-values so that the probability of observing at least one significant result due to chance remains below the desired significance level. The Benjamini-Hochberg (BH) adjustment is a statistical method that provides a p-value answering the following question: what would be the percentage of false positives if all the genes with adjusted P-values threshold were considered significant? For example, by setting a threshold of 0.1 for the adjusted P-values, we consider a 10% false positive rate, or false discovery rate (FDR) as acceptable, and identify the genes that are significantly expressed by considering all the genes with adjusted P-values below this threshold.

We calculated the cutoff value for FDR by using the Matlab command `mafdr`. This command supports the BH method as well as other multiple testing correction approaches. Note in Figure 2S-a that the profile of p-values for exons and introns are different. After setting an FDR of 1% for both exons and introns and obtaining a p-value cutoff, we selected the lower of the p-value cutoffs. Because dispersion of counts in RNA-Seq is assumed to be dependent on mean counts, we performed an additional check in order to select the most conservative false positive rate. We divided the set of genes with significant p-values into three sets: set 1 with upper tertile (the upper 1/3 of counts), set 2 with midrange counts (mid tertile), and set 3 with lower counts. In each case, we set the false positive rate at 1% and noted the p-value at which this false positive rate is achieved. We used the lowest p-value among the three cases (after adjustment for subsets) to guarantee a false positive rate of 1% or lower.

In addition to the MATLAB function "`nbintest`", we used the function "`mafdr`" in order to identify significance with *p* values less than 0.001, and initially limited the set to absolute log2 fold changes greater than unity. The command is: `fdr = mafdr(pvals,` 'BHFDR', 'true');

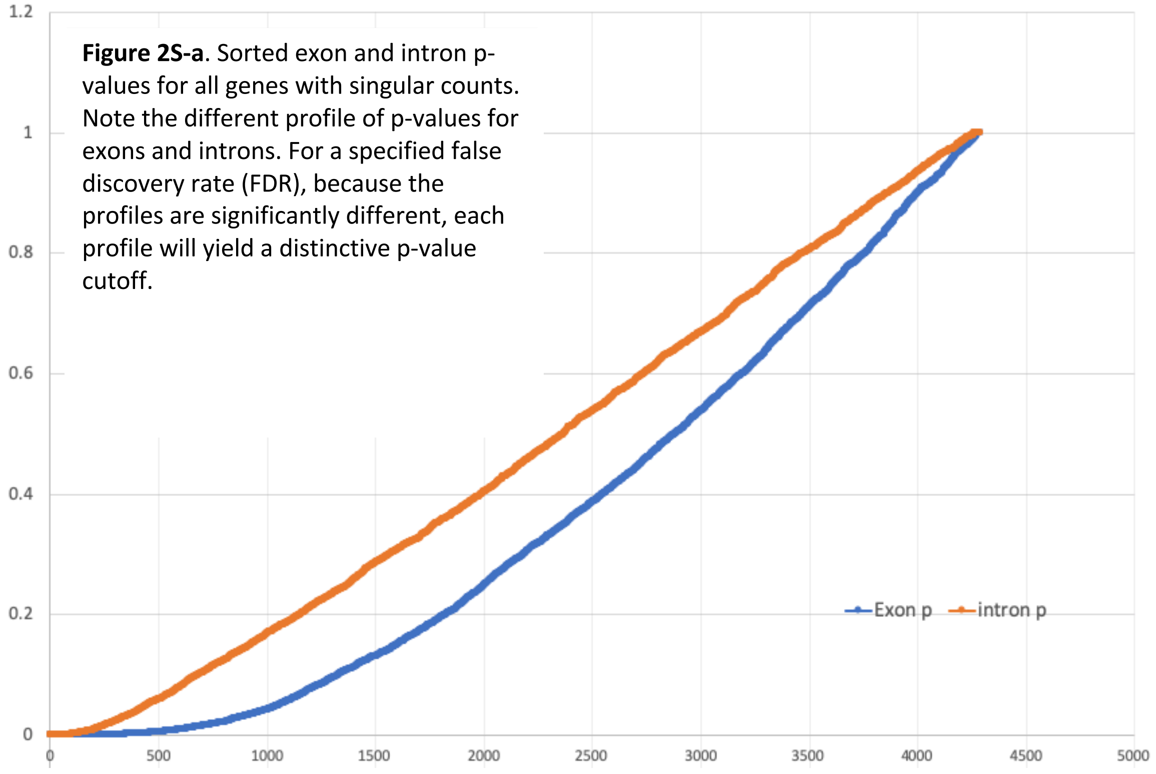

Figure 2S-a. Sorted exon and intron p-values for all genes with singular counts. Note the different profile of p-values for exons and introns. For a specified false discovery rate (FDR), because the profiles are significantly different, each profile will yield a distinctive p-value cutoff.

Section S4. Clustering details

Preparation

For clustering analysis, we normalized the exon and intron count levels for each gene by proportionally adjusting the counts to the lowest exon or intron expression level as outlined in the manuscript. For the exon-only analysis, we performed a log2 transform on the normalized values. For the exon:intron ratio analysis, we adjusted exon and intron counts, computed the ratio and performed a log2 transform. We took care to correct for cases where the lowest

expression level was equal to zero and used the Pearson correlation coefficient as the distance measure in the K-Means algorithm in both exon-only and exon:intron ratio analysis.

Comparison of cluster patterns

We initially compared the clustering results from the exon-only values and exon:intron ratio data for the 35 healthy individuals in Figure 1 (main manuscript). In order to compare the exon-intron ratio cluster profiles observed in Figure 1 with the Control data presented in the archived data set (see manuscript for reference), we used genes common to both data sets in order to arrive at the same number of total genes for comparison. The clustering results shown in Figure 3S, panels A and B depict two cluster profiles from two distinct sets of control data for the 3934 common genes. The number of genes in each cluster is different but the image size for each cluster has been adjusted to the same size for the clarity of presentation. The four exon-intron clusters identified in panel A contained 968, 1412, 710, 844 genes (in order, left to right in panel A), whereas the clusters in panel B for the exon-intron clusters in the Mo et. al control data had 1009, 1491, 701, 733genes (in order, left to right in panel B).

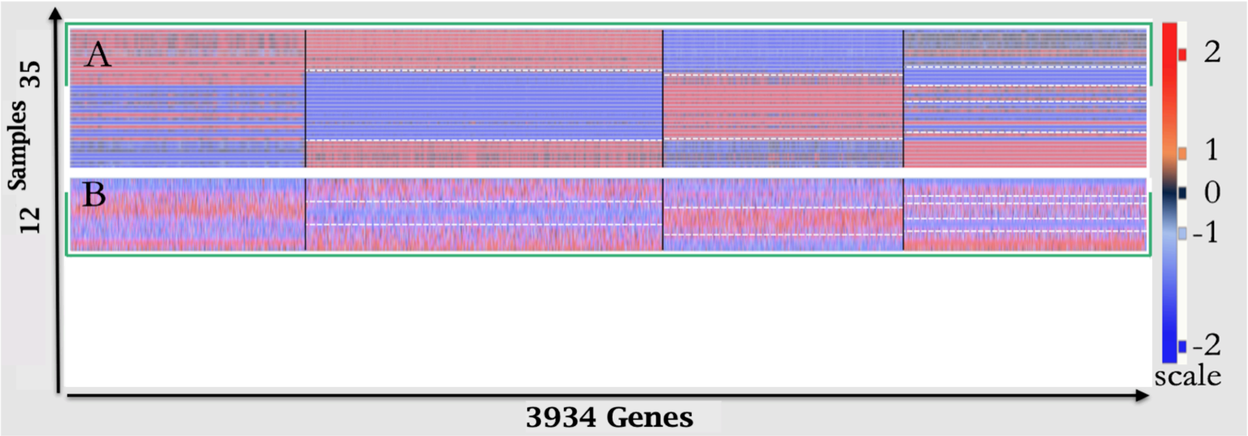

Figure 3s – Exon:intron Clustering results for the two control data sets. Two panels illustrate the clustering results from the exon:intron ratio clustering for the 35 controls (panel A) shown in the manuscript and the 12 controls in the Mo et. al. polyarthritic data set (panel B). The analysis of both data sets yielded 4 clusters with similar clustering patterns across samples (e.g A vs B vertical axis profiles). The clustering patterns in the far left panel (968 genes for A and 1009 genes for B) exhibit somewhat dissimilar patterns which may be due to the small size of the control set (12 samples) in the archive data.

Section S5. Correlation Coefficients and regression

Correlation coefficients. We evaluated both the Pearson and Spearman rank correlation coefficient in order to investigate the relationship between exon and intron counts. In practice, discovering nonlinear data relationships remain a research problem. Therefore, we focused on a practical class of nonlinear correlations represented by Spearman rank correlation. As shown in the figure 4s, the two correlation values for the 35 in-house controls were correlated – indicating that the relationship could be captured effectively by the Pearson correlation coefficient. We used Pearson correlation coefficient in all subsequent analysis.

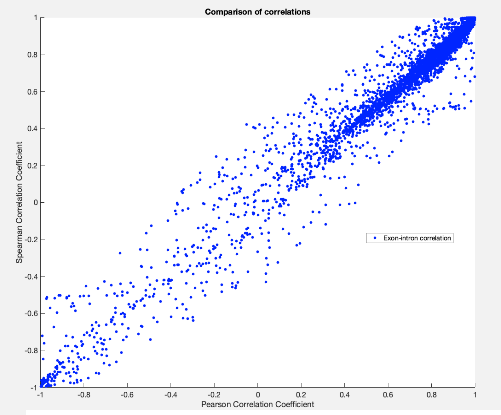

Figure 4s – The relationship between Pearson correlation coefficients and Spearman rank correlation coefficients for the exon:intron data of 35 in-house controls.

Section S6 . Exon and intron variability rank ordering

We tested gene sets with the lowest (top panel) and highest variability (bottom panel based on their GINI scores), using the ranked window concordance (RWC) test. Note that RWC is a simpler measure compared to ARI (used in the manuscript) and it measures the fraction of common elements (e.g. exons and introns from the same gene) between two ordered sets as a function of the number of elements selected. The selection is performed based on the order of elements in a set. Selection order can be either increasing (first element to last element), or decreasing (last element to first element). It is a simplified version of Kendall's tau correlation for windowed data (different size lists). It is an unweighted measure of similarity between two lists. We used gene lists corresponding to the lowest variable exon expression level in windows ranging from 100-1000 genes. We repeated the same analysis for the highest variability expression genes using the same window sized.

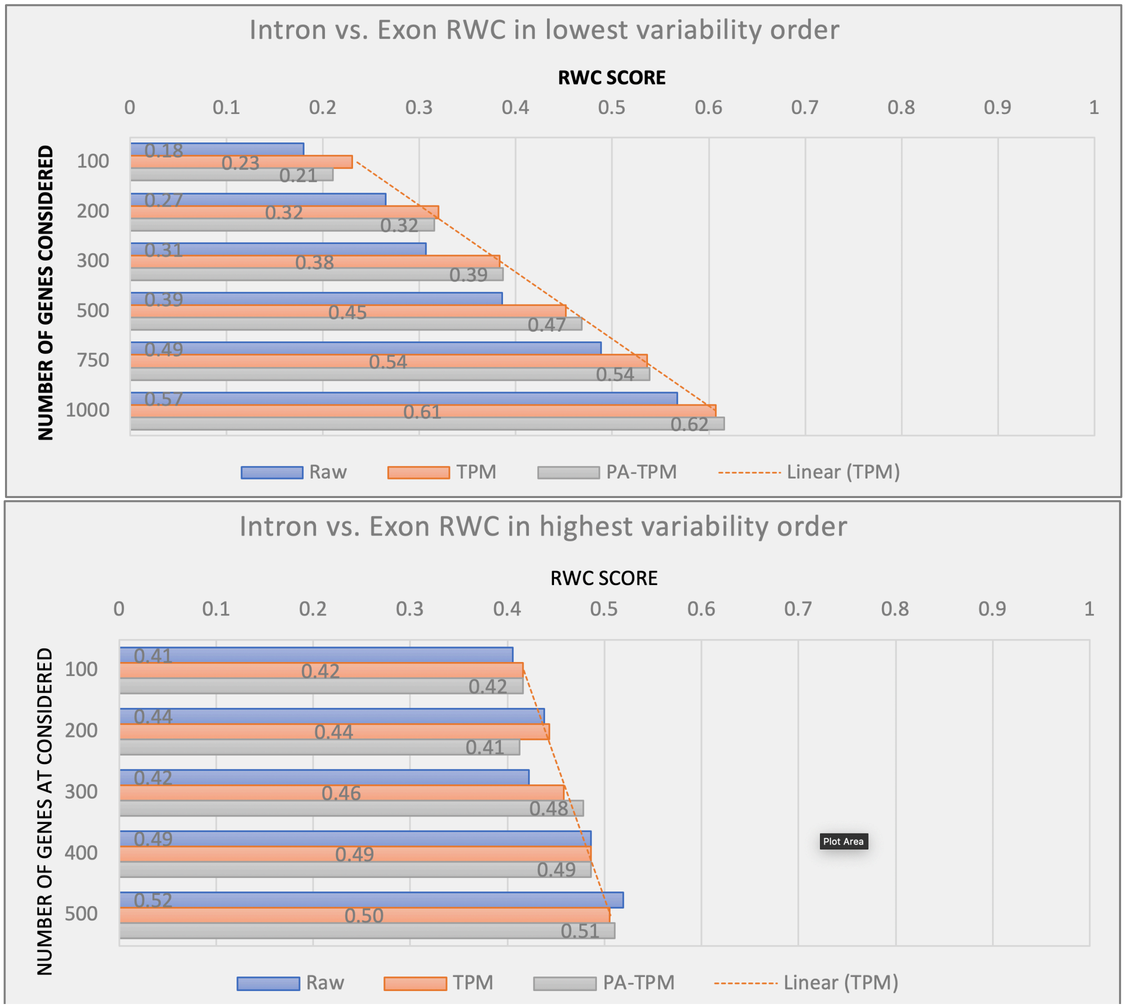

Figure 5s. **Rank window concordance of RNA-Seq expressions for exons and introns.** Ranked concordances as a function of variability – for genes with lowest GINI coefficients, as well as highest GINI coefficients – demonstrates that exon and intron expression variability is substantially different. As illustrated in the figure, TPM adjustments, and by ERCC calibration (Proportionally adjusted (PA), have a minimal impact on RWC scores.

Section S7. String-db and pathway search results

All gene lists identified in this study were submitted to the Strings-db (version: 11.0) using the web interface for "Multiple proteins" with the organism selected as homo sapiens. Default matches against String-db's name database were accepted. Default settings were used for parameters.

String db search results for the 25 genes identified by standard gene expression calculations .

From the list of 25 genes, 22 genes were identified as annotated genes by the Strings-db database Figure 6s) .

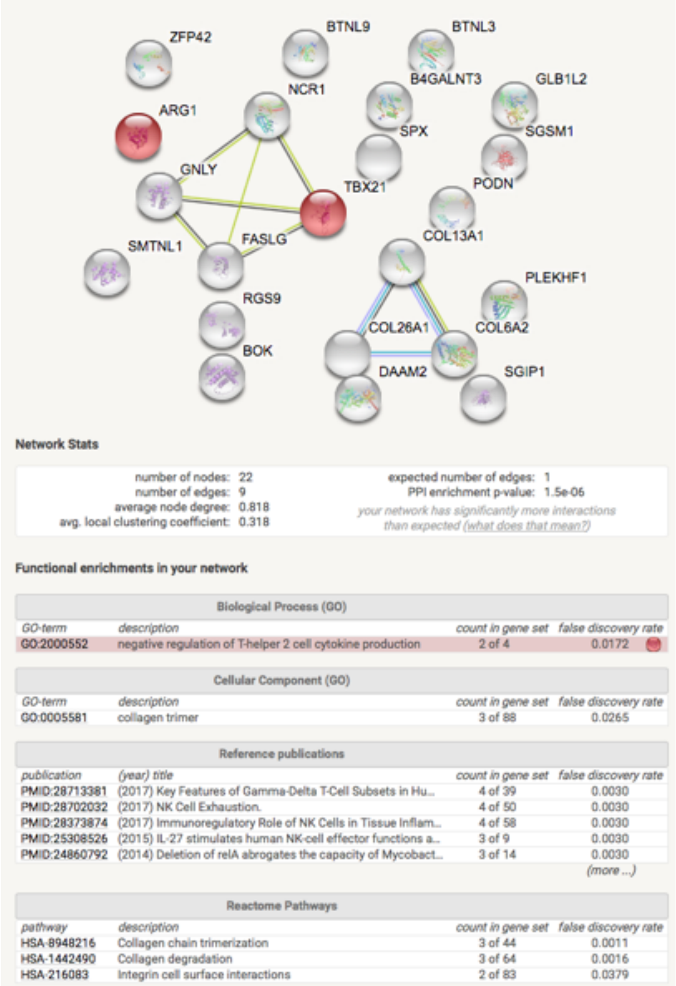

Figure 6s - Strings-db analysis of a list of 25 genes identified with methods commonly employed for the identification of differentially expressed genes. . PPI enrichment was ≥ 1.5 e-06 and no significant biological pathways were identified.

Section S8. Data

Raw data for the singular counts is contained in six comma-separated-value files (.csv). Two files contain the data for the 35 in-house controls (inhouse_exon.csv, inhouse_intron.csv). The second set of files contain the control and case data from the Mo et. al study. The control data files are MoControl_exon.csv and MoControl_intron.csv, and the polyarthritic case files are MoCase_exon.csv and MoCase_intron.csv.

Section S9. Additional procedures for evaluating the RNA-Seq data

Exon-intron expression level is not a strong factor in correlations between exons and introns.

We checked to see if the exon-intron expression level is a primary determining factor in exon-intron correlations. We plotted exon-intron correlations against expression level for each gene and considered the range and correlations present at various expression levels. There is little to no impact from expression levels on the observed correlations, Figure 7s.

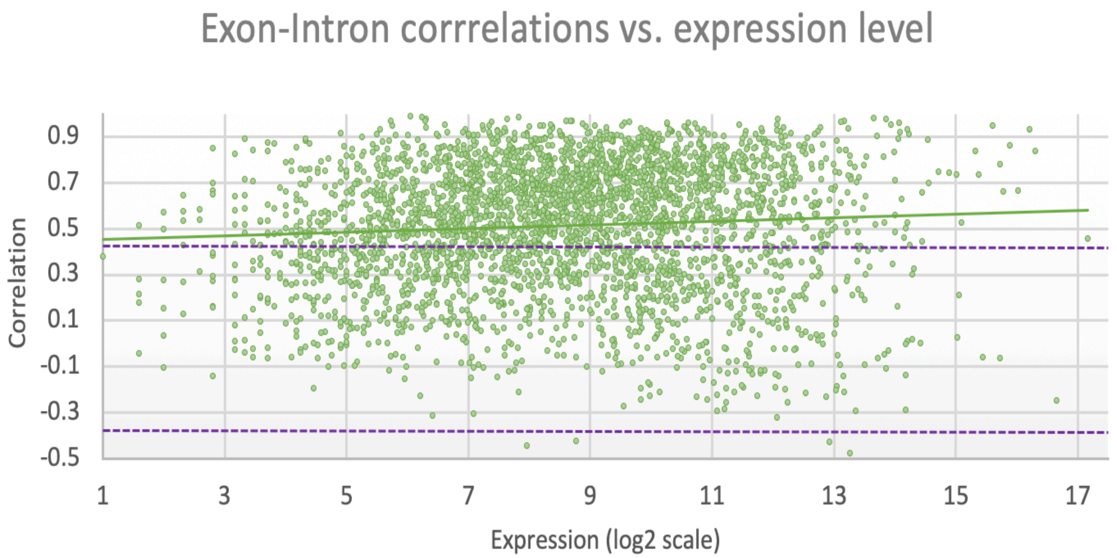

Figure 7s. Correlation as a function of exon expression level. The coordinate of each gene dot on the figure is depicted as an exon-intron gene pair . For clarity, only data for genes with correlation between -0.5 and 0.95 are shown. There is a nearly uniform distribution of data points across 8 (log 2) scales, suggesting that correlation is not a function of expression level. The figure recapitulates the range of exon-intron correlations shown in Figure 2 in the manuscript.

Nature of change in Exon and intron GINI scores across control and case conditions. To test the hypothesis that two empirically calculated Gini coefficients G_1 and G_2 are statistically the same for two populations, we used the test statistic $(G_1 - G_2)/S$, where $S = \sqrt[2]{\sigma_{G_1}^2 + \sigma_{G_2}^2}$ is the square root of the sum of calculated scatters based on empirical data [For details, see Mills, J. A. and S. Zandvakili (1997). "Statistical inference via bootstrapping for measures of inequality", Journal of Applied Econometrics, Vol. 12, 133–150.]. We then used a bootstrap approach on a subset of 400 samples and compared the mean GINI ratios and the significance level calculated by using the following resampling procedure. A sample was generated by resampling with replacement from each of the two populations. Then, the bootstrap statistics was computed by using $(G_1 - G_2 - G_1^* + G_2^*)/S^*$ - the * indicates the bootstrapped result. Our working assumption was that samples from the population are uncorrelated and therefore no correction (through the covariance matrix) to the denominator (statistics) is needed. This correction can be implemented but it was found to have little impact. In the final step, we resampled pairs of observations to generate bootstrap samples. In 100% of the 400 cases in which the significance was less than 0.001 (12 cases from the 400), the GINI ratio of control to case was 2 or higher (or 0.5 or lower). Therefore, we used the criteria of 2x or higher as the basis for selection (or 0.5 or lower). This procedure was necessary in order to obviate the lengthy and expensive bootstrap calculations for nearly 15000 genes in 3 data sets. We note that the time for each bootstrap was 24 minutes (average), and the 400 cases where simulated 3 times for a total time of 5 days on a multicore processor. The subsequent examination of the results for the 3 simulation runs for each 400 samples was also necessary.

Exon and intron GINI values are generally stable across treatment groups (control vs. case) and do not change significantly (Figure 8s). For a few genes, there is a large change in the variability of either exon or intron GINI values. The lines of 2x change (dashed lines) approximate the 99% confidence interval indicating that these changes are highly unlikely to have occurred by chance alone. The 99% confidence interval was established through simulated computational studies.

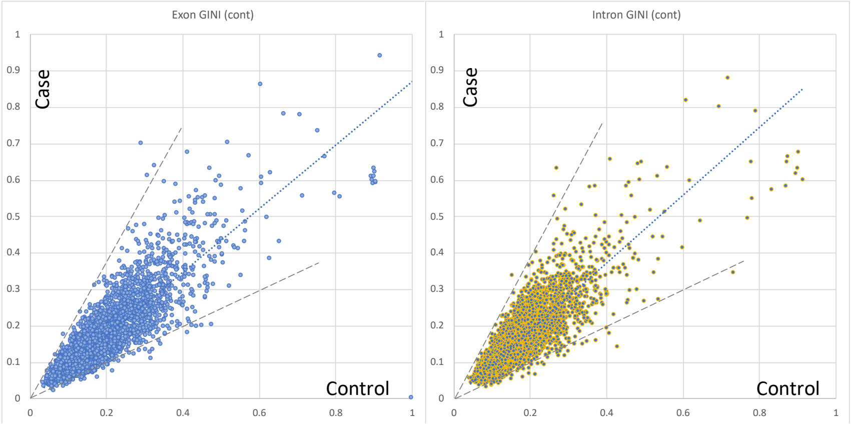

Figure 8s - Control vs. Case GINI scatter plot. X-axis is the GINI value for control expression data (blue outline circles: exon GINI, orange outline circles: intron GINI). Center dotted line in each figure is the trend line (linear regression). The two outer ~~wo~~ dashed lines around the center dotted line represent the 2x change lines. Genes outside of these dashed lines show a 2x GINI change (exon or intron) in control vs. case samples.

Correlation of Ratio variables in the exon:intron clustering study when intron expression is random. We performed this simulation in order to evaluate the exon/intron ratio correlation vectors under the assumption that intron values are simply "noise". We used the exon values from our study and generated intron values to simulate a from a randomly generated (uniformly distributed) noise process as illustrated in figure 9s. After dividing the exon by the intron value, we calculated the correlation of the exon/intron ratio vector with 200 other vectors from genes that were randomly selected. We recorded the average correlation from 320 simulation trials. The resulting average over all the trials demonstrated that these correlations are generally very low and rarely rise above 0.2. In other words, if intron values were simply due to noise, K-means clustering would not have been able to form a small number of well-correlated clusters.

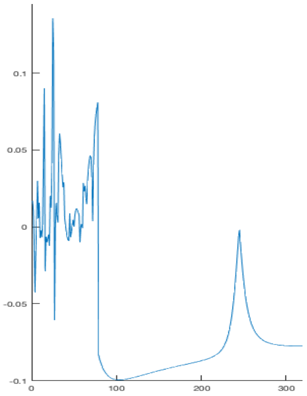

Figure 9s - Simulated average correlation of exon/intron ratio vectors.
